# Supplementary material for: Real-time holographic lensless micro-endoscopy through flexible fibers via fiber bundle distal holography
Source: Nat Commun. 2022 Oct 13;13:6055. doi: 10.1038/s41467-022-33462-y (PMC9563069; doi:10.1038/s41467-022-33462-y)
Supplement: Supplementary file 1 — Supplementary Information [file 41467_2022_33462_MOESM1_ESM.pdf]

SUPPLEMENTARY INFORMATION

Section S1: Theoretical resolution and field-of-view analysis

Resolution

Collecting the intensity through MCF limits the system resolution due to both the diameter of the fiber, D, and the maximum accepted numerical aperture NA_{max} . Each core fundamental-mode NA will limit the collected light to a maximum NA of NA_{max} for objects that are at a distance closer than: $z_o \approx \frac{D/2}{NA_{max}/n} - z_m$. For objects at larger distances the NA will be limited by the bundle diameter to: $NA_{eff} \approx \frac{nD/2}{(z_o+z_m)}$. The theoretical diffraction-limited resolution of the system can thus be summed up in the two regimes as follows:

$$\sigma_x = \frac{\lambda}{NA_{eff}} = \begin{cases} \lambda \frac{1}{NA_{max}} & z_0 < \frac{Dn}{2NA_{max}} - z_m \\ \lambda \frac{2(z_m + z_o)}{Dn} & z_0 \geq \frac{Dn}{2NA_{max}} - z_m \end{cases} \qquad \text{(S1)}$$

In practice, NA_{max} may be different than the maximum NA of the MCF cores due to the digital Fourier-filtering (i.e. interpolation) made in order to suppress aliased replicas resulting from the spatial sampling by the fiber cores (see Methods).

To validate this theoretical estimation and characterize the experimental system resolution, we performed a knife-edge measurement, where a sharp-edged reflective target was imaged (Fig. S3 A). The Point Spread Function (PSF) was calculated from the spatial derivative of the reconstructed target image for each image row, as shown in Fig. S3 A, B. The results for the obtained resolution as a function of the target distance, shown in Fig. 3J, are the FWHM of the calculated PSF averaged over the image rows. These are in very good agreement with the theoretical prediction, given in Eq.S1. The error-bars of Fig. 3J are the standard deviation of the PSF FWHM, calculated at 100 different rows of the image.

Field of View

The theoretical Field of view (FOV) limitations of the system can be estimated from geometrical considerations: First, the object must be illuminated by the illumination field, which has an NA of the first mode of the MCF core (NA_1), i.e. $FoV \approx 2NA_1(z_o + z_m)/n$. For diffuse reflecting objects, this is the only limitation, and the spatial field of view can theoretically be larger than the MCF diameter, D. However, for specular targets that are parallel to the fiber facet, the reflection angle is similar to the incidence angle of the illumination light (neglecting the effects of diffraction), and light will be collected by the MCF only from reflectors that are located at a $FOV < \frac{D}{2}$. To sum up the theoretical considerations predict a FoV that is:

$$FOV = \begin{cases} \frac{D}{2} & for\ specular\ targets \\ 2NA_1(z_o + z_m)/n & for\ diffuse\ targets \end{cases} \qquad (S2)$$

As explained in the following section, the mirror distance in our experiments, z_m, is chosen such that $4NA_1 z_m \geq D$, resulting in $FOV \geq D/2$ for both specular and diffusive targets.

To validate the theoretical estimation and characterize the experimental system FoV, we characterized the FoV of the experimental system using a mirror as the imaged object. The mirror was reconstructed using digital back-propagation, but without normalizing by $|E_{illum}|$, leaving a Gaussian-like intensity profile. The diameter of the reconstructed intensity envelope can be used as a measure for the system FoV. As a figure of merit, we calculated the $1/e^2$-width of the reconstructed intensity profile. The result of these FoV measurements performed at multiple distances, are displayed in Fig.3I. They demonstrate an almost constant FoV at all distances, as theoretically expected. The practically useful FoV can be larger than this estimate since the exact definition of the FoV is dictated by the required signal to noise ratio (SNR), which is dependent on the illumination intensity and target reflectivity. In our experiments for imaging a reflecting USAF target using a $D = 650\mu m$ MCF, details of the target are clear at a FoV exceeding $300\mu m$ at all distances as shown in Fig. S3 C.

Each data point in Fig. 3I is the average $1/e^2$-width of the reconstructed image of the mirror, averaged at 6 different angles in a single reconstructed image, at intervals of 30 degrees. The error-bars in Fig. 3I are the standard deviation of the $1/e^2$-width at these 6 angles.

Section S2: Choice of optimal system parameters

Distal mirror distance

To choose the mirror distance, z_m, both the phase variation and the intensity profile of E_{ref} must be considered. First, the phase of the field is a spherical one, with a curvature of $2z_m$: $\phi(E_{ref}) = \frac{2\pi n}{\lambda 4 z_m}(x^2 + y^2)$. To ensure a well-defined unvarying reference phase at each core, we demand that $d\frac{\partial}{\partial x}\phi(E_{ref}) \ll \pi$ over the entire distal facet. Differentiating the above expression for $\phi(E_{ref})$, and plugging $x_{max} = D/2$ results in the following demand for the mirror distance:

$$z_m \gg \frac{Ddn}{2\lambda} \qquad (S3)$$

In addition, the intensity profile must be wide enough to cover the entire fiber, i.e., requiring that $z_m \geq \frac{nD}{4NA_1}$. The first requirement (eq. S3) automatically fulfills the latter, since $NA_1 \leq \frac{\lambda}{2d}$. For the Schott fiber used throughout the article, D=500μm, NA_1=0.2, used in optical oil the required distance is $Z_m > 1200mm$.

Distal mirror reflectively and reference- and signal-arms powers ratio

Three important system parameters affect the SNR of the measurements and can be determined by the demand to maximize the SNR of the system. First, the reflectivity of the partially reflecting distal mirror, R, i.e., the power reflection coefficient. Second, the total illumination power emerging from the distal facet, P. The third parameter, A, is the fraction of the total illumination power which is in the reference arm. For the sake of simplicity, no absorption is considered, and an object with a total effective power reflection coefficient of $R_o \ll 1$ is considered. Where R_o also takes into account the power lost to propagation and effective reflective area of the object. For further ease of notations, we denote the power ratio in the object arm $B = 1 - A$, and the power transmission coefficient of the mirror $T = 1 - R$.

To analyze the optimal SNR, one may consider the signal collected by a single core. In each single core, four fields are superimposed, as described in "Suppression of spurious reflections" section. Using the same notations, we analyze the expected amplitude of each of them:

$$\begin{aligned} |E_{R,m}| &= \sqrt{PA}\sqrt{R} \\ |E_{R,o}| &= \sqrt{PA}\sqrt{T^2 R_o} \\ |E_{I,m}| &= \sqrt{PB}\sqrt{R} \\ |E_{I,o}| &= \sqrt{PB}\sqrt{T^2 R_o} \end{aligned} \qquad (S4)$$

Where $E_{R,m}$ and $E_{\mathrm{R,o}}$ are the reflections of the reference beam from the mirror and object, respectively. $E_{\mathrm{I,m}}$ and $E_{I,o}$ are the reflections of the illumination beam from the mirror and object, respectively.

The desired signal is the interference term between $E_{R,m}$ and $E_{I,o}$, while the constant background is proportional to all four intensities. Assuming the dominant source of noise in the camera detected signal is shot-noise from the strong background, as is commonly the case when using sCMOS cameras, the SNR in a single frame will be:

$$SNR \propto \frac{|E_{R,m}E_{I,o}|}{\sqrt{E_{R,m}^2+E_{R,o}^2+E_{I,m}^2+E_{I,o}^2}} = \sqrt{P}\cdot\sqrt{AB}\cdot\frac{\sqrt{RT^2R_o}}{\sqrt{R+T^2R_o}} \qquad (S5)$$

The SNR is thus a product of three terms: the first is the total illumination power, that is limited in most practical applications by sample damage. The second term $\sqrt{AB} = \sqrt{A(1-A)}$ is maximized by setting $A = B = 0.5$, i.e., setting the powers in the two arms to be equal with a balanced MZI. Lastly, the third term in eq. S5 is maximized by setting R and T to fulfill:

$$\begin{aligned}\frac{R^2}{T^3} &= \frac{R_o}{2} \\ \Rightarrow R &\approx \sqrt{R_o/2}\end{aligned} \qquad (S6)$$

The last approximation is valid only assuming $R \ll 1$, which is indeed the case when considering weakly-reflecting objects. For example, for $R_o = 0.02$, equation S6 yields $R \approx 0.1$. In our experiments R is determined in the mirror fabrication process. For the results shown throughout this article, $R \approx 0.12$ was achieved by evaporating 10nm of titanium on a microscope slide. To fine-tune the power ratio of the two arms, A, a polarizing beam-splitter along with two half-wave-plates was incorporated into the setup, as explained in the "Experimental design" section.

Fiber parameters

Commercial MCFs are characterized by several important parameters: the pitch, p, the single core NA, the number of modes in a single core, the total fiber diameter, D, and the intracore crosstalk. To use FiDHo, the correct fiber and mirror distance must be chosen. First, to maximize resolution, a large NA_{max} is needed, as achieved with larger core sizes. In addition, increasing the bundle size D will increase both NA_{eff} at larger angles and the FOV for weakly scattering targets, at the price of a larger footprint. Smaller pitch will improve resolution, since less spatial interpolation will be needed on the fiber facet. Lastly, to reduce bending sensitivity and to increase resolution, a fiber with minimal crosstalk should be chosen, while maintaining a reasonable fill factor. The fiber and laser used throughout this article is a subset of Schott 1533385,$D = 1100\mu m$, $NA_{core} \sim 0.2$, $\lambda = 640nm$ where only $D = 650\mu m$ was used. In all experiments, a mirror distance of $z_m = 2000\mu m$ was used. An additional fiber, Fukijura FIGH-06-300S, $D = 270\mu m$,$NA_{core} \sim 0.3$, was used only in figure S4.

Similar to fiber-bundle based imaging schemes, a lower inter-core crosstalk is important to the FiDHo approach. Since in FiDHo the fiber-bundle is used both in the illumination step and the collection step, in both reference and object arms, the influence of the inter-core crosstalk can be divided into several effects:

1. In the illumination path, a high crosstalk will result in leakage of the light to adjacent cores, thus creating a complex interference pattern in the reference and object illumination beams. The resulting effect is twofold:
   a. The reference field is assumed to be the diffraction of the fundamental mode of a single core. Intercore crosstalk will result in an unknown reference field that is also varying and very sensitive to fiber bending.
   b. The object illumination would have dark areas due to destructive interference. In addition the object will be illuminated by an unknown spatially varying, and bend-sensitive phase, which would not allow true phase reconstruction.

2. In the collection path, similar to conventional fiber-bundle based imaging approaches, a large intercore crosstalk will cause the signal to leak to adjacent cores, reducing the resolution of the measured hologram image, and creating coherent interference between the different cores, that would be bend sensitive.

Section S3: Digital filtering of MCF pixelation

The distal holography approach is based on holographic recording of the field at the distal facet. However, since the field hologram is not sensed directly but is effectively sampled the fiber cores, having a limited fill-factor due to the core-to-core pitch being larger than the mode diameter, sampling-induced artefacts (resulting from aliasing in spatial frequency space), appear in the reconstructed field. To achieve the high image quality of FiDHo, these sampling-induced artefacts need to be suppressed. Here, we obtained the required suppression of these artefacts by a combination of imaging and illumination geometry and natural filtering of the MCF cores, which attenuated high spatial frequencies, in addition to digital filtering in post-processing. In this section we provide a mathematical and numerical analysis of the sources for the sampling-induced artefacts, and of the effectiveness of the approaches we have implemented to suppress them.

We first briefly present and explain the experimentally observed artefacts, as displayed in Supplementary Fig.S2a-c. In our experiments with a commercial MCFs produced by Schott, cores are located on a hexagonal grid with a core-to-core pitch, $p = 8\mu m$ (Fig.2A). Thus, the incident field hologram is effectively sampled at a spatial sampling frequency, $f_s=1/p$. As result of the convolution theorem, in the Fourier (angular frequency) domain, this spatial domain sampling results in generation of replicas of the object angular spectrum at shifts of $\Delta f = mf_s$, where m=±1,±2,… (Fig.S2B). If the object plane is located at a distance, z, which is effectively at the far-field of the MCF, these aliased replicas will limit the FoV to $\sim \lambda z/p$ [46]. If the object is located at shorter distances from the fiber, the angular frequency artefacts will results in object replicas inside the imaged FoV (Fig.S3C).

To yield the following mathematical analysis more accessible and without loss of generality, we perform the analysis in one dimension (1D). Extension to two dimensions (2D) is straightforward.

Consider an electric field, $E_{signal}(x)$, being measured through a 1D array of waveguides spaced evenly with a pitch p, representing the MCF cores. Each waveguide can guide light with a complex amplitude that is proportional to the overlap-integral of the electric field that impinges upon it with a certain mode M_d, of typical mode-field-diameter d. Therefore, the measured field on the other side of the array will be:

$$E_{meas}(x) = \Big(\big(E_{signal}(x) * M_d(x)\big) \cdot comb_p(x)\Big) * M_d(x) \qquad \text{(S7)}$$

Where $*$ symbols a convolution that realizes the overlap integral with the fiber mode, $comb_p$ is the Dirac-comb composed of delta-functions spaced at intervals of p.In the angular frequency domain, this sampled field is given by:

$$\tilde{E}_{meas}(f_x) = \Big(\big(\tilde{E}_{signal}(f_x) \cdot \tilde{M}_{1/d}(f_x)\big) * comb_{1/p}(f_x)\Big) \cdot \tilde{M}_{1/d}(f_x) \qquad \text{(S8)}$$

Where the convolution theorem was used. Assuming that M_d is a gaussian-like function of typical width d we can assume $\tilde{M}_{1/d}$ to be of typical width $\frac{1}{d}$. We note that in an MCF the core diameter is smaller than the core spacing: $d < p$.

Several conclusions can be drawn. The overlap with the fundamental mode is an effective low-pass filtering in the spatial-frequency domain, with a cutoff frequency of approximately $|f_x| = \frac{1}{d}$, as is expected from the finite NA of the fundamental mode.

However, since the filtered fiels, $\tilde{E}_{signal}(f_x) \cdot \tilde{M}_{1/d}(f_x)$, is then convolved with a dirac-comb, aliasing occurs (see Supp. Fig. S8). Thus, replicas of the measured signal spatial spectrum will appear in intervals of $\Delta f_x = \frac{1}{p}$. Aliasing of frequencies that are well sampled (fulfilling the Nyquist sampling rate, i.e. $|f_x| < \frac{1}{2p}$) appears at frequencies that are higher than

$f_{cutoff} = \frac{1}{2p}$, and thus they can be removed by low-pass filtering. The result of such filtering is presented in Supplementary Figure S2(D-F). Note that in the spatial domain, the frequency-domain lowpass filtering is a convolution with a blur kernel of size 2p, i.e. a Fourier-interpolation of the sampled field (Supplementary Figure S2d).

Note that if $\tilde{E}_{signal}$ contains high spatial frequency components of $|f_x| > \frac{1}{2p}$ that remain after the physical filtering of the fiber mode, they will be aliased to the passband of the lowpass filter ($|f_x| < \frac{1}{2p}$) and will result in image artefacts, which may be reduced by advanced image reconstruction algorithms. Importantly, in our experimental realization such high spatial frequencies are of low amplitude, due to the intentionally sufficiently long minimal distance between the object and the fiber distal facet.

To illustrate the above analysis, Supplementary Figure S8 displays a numerical example for the results of sampling, aliasing, and digital filtering in one dimension.

Section S4: Analysis of the effects of higher-order modes in each core

As each core of the commercial MCFs used in our work supports several transverse modes, in our experiments care was taken to excite only the fundamental mode by the illumination and reference beams. However, such excitation is not perfect, and in addition fiber bending and manufacturing imperfections may couple the beams injected in the fundamental mode to higher modes. This requires additional care in the measurements and analysis to the possible contribution of higher order modes of each core to the image formation. The most important and very effective experimental measure to minimize the contribution of the higher order modes is our use of the short coherence length source to time-gate the interference with the fundamental mode, which temporally arrives before the higher order modes. This was proved to be sufficient to reduce the contribution of higher order modes to a negligible measure. To illustrate this, below, we experimentally characterize the higher order modes in our experiments, and numerically analyze the potential effects of higher order modes on the reconstructed images.

Experimental measurements

To experimentally characterize the temporal delay and relative amplitude of the second transverse fiber mode, we performed a measurement of the total energy of the recorded hologram as the relative delays between the two interferometer arms (the 'object illumination' arm and the 'reference' arm) is scanned, for a target object placed at a distance of $200\mu m$ from the distal mirror. The results of this scan are displayed in Fig S5.A.

As expected, the measured scan displays three dominant peaks (i,ii,iii): the most dominant peak (i) appears at zero relative delay, and is dominated by the interference of the strong reflection from the distal mirror in one arm with the same reflection in the second arm. In addition, it contains the interference of the illumination field reflected from the object with the reference field reflected from the object. As we explain below, when a second mode is present, additional mirror-mirror and object-object interreferences are contributing to the hologram due to the second mode (see Fig.S5E and explanation below). The second time-gated interference peak (ii) is the desired interference between the fundamental mode illuminating the object and the fundamental mode reference beam reflected from the mirror, used for imaging in our experiments. This peak appears when the delay is set to the object distance: $\tau = 2z_o/(c/n)$. The third time-gated interference peak (iii) is the result of the reflections from the second mode in the illumination arm with the fundamental mode in the reference arm (see temporal diagram in Fig.S5E).

In accordance, the spatial shape of the holograms measured at these three delays reveal the nature of the different fields that take part in each interference: for (i) the hologram reveals the distal mirror flat-phase reflection (Fig.S5B); for (ii) the hologram reveal the object reflected field on the facet (Fig.S5C), which is used for image reconstruction; for (iii) the hologram reveals the second-order mode illumination of the distal mirror, as measured by the fundamental mode (Fig.S5D). Most importantly, this experiment displays how the short time-gating allows not only to filter the strong mirror reflection, but also to effectively filter the unwanted contributions of the second (and higher) modes.

To clarify the different expected contributions to these three interference peaks (i-iii), we have plotted in Fig.S5E a sketch of the reflections from the distal mirror and object for the first two modes in each arm, for the three different delays (i-iii).

As in Supplementary Section S2, we denote the fields of the reference arm by subscript (R), the fields from the illumination arm by subscript (I), the reflection from the distal mirror or object are denoted by subscripts (m,o) respectively. The first and second mode are denoted by superscript (1) and (2). The fields generated by the reference arm are thus: $E_{R,m} = E_{R,m}^{(1)} + E_{R,m}^{(2)} + E_{R,o}^{(1)} + E_{R,o}^{(2)}$, and the fields generated by the illumination arm are thus: $E_{I,m} = E_{I,m}^{(1)} + E_{I,m}^{(2)} + E_{I,o}^{(1)} + E_{I,o}^{(2)}$. As result of the short time-gate, the fields that contribute to each of the interference peaks, are only those that temporally overlap, as marked by the dashed vertical lines in Fig.S5E. At the desired delay for imaging (ii), the measured hologram is the result of the interference of the fundamental-mode reflection from the distal mirror of the reference beam with the reflection of the fundamental mode of the illumination beam from the object, as desired: $E_{I,o}^{(1)} E_{R,m}^{(1)}$. The relatively weak interference of the second mode illuminating the mirror and object ($E_{I,o}^{(2)} E_{R,m}^{(2)}$) is coherently added to the strong hologram of the first mode. The interference due to the second mode is very small in magnitude since its amplitude is squared in the recorded hologram (both reference and illumination fields are excited weakly in the higher order mode). From the measured experimental trace (Fig.S5A) we estimate that the energy in the second order mode is approximately 2% of the fundamental mode energy.

Numerical investigation of higher-order mode impact on reconstructed images

In the previous section, we have shown that the experimentally measured hologram at the optimal time-delay is the sum of two interference terms: $E_{I,o}^{(1)} E_{R,m}^{(1)} + E_{I,o}^{(2)} E_{R,m}^{(2)}$. The first is the desired hologram generated by the fundamental mode, and the second is a significantly weaker undesired hologram that is generated by the second mode. Experimentally, the weak undesired hologram contribution is difficult to measure and separate from other noise and background contributions. Thus, in order to complement the experimental investigation and characterization of the higher-order modes, we have performed a numerical simulation where we study the impact of the contribution of the second mode on the reconstructed image.

The results of this numerical investigation are displayed in Supplementary Figure S6. The results are obtained from a simulation based on digital angular spectrum propagation, which is performed separately for each mode of the MCF core. The holographic recorded field is simulated in several steps: (i) the illumination field from a selected single mode ($j = 1,2, \ldots$) from a single core at the MCF distal facet is propagated to the object plane; (ii) the field illuminating the object is multiplied by the 2D object reflection function; (iii) the reflected field is propagated back to the MCF distal facet to produce the object field from the illumination arm at the distal facet for each mode, j: $E_{I,o}^{(j)}(x,y)$; (iv) the same steps are performed for all the fiber modes studied when replacing the object reflection function and distance by the distal mirror reflection, to simulate the reference field at the distal facet for each mode: $E_{R,m}^{(j)}(x,y)$. The simulated holographically measured fields are then calculated for each mode by:

$$E_{holo}^{(j)}(x,y) = \left(E_{R,m}^{(j)}(x,y)\right)^* E_{I,o}^{(j)}(x,y) \qquad (S9)$$

Following (Eq.1), the reconstructed object field image is produced by normalizing the holographic recorded field by the expected diffraction of the reference mode $\left(E_{R,m}^{(1)}(x,y)\right)^*$, followed by back-propagation:

$$O^{(j)}(x,y,z_{prop}) \propto \mathcal{P}_{-z_{prop}}\left(\frac{\left(E_{R,m}^{(j)}(x,y)\right)^* E_{I,o}^{(j)}(x,y)}{\left(E_{R,m}^{(1)}(x,y)\right)^*}\right) \qquad (S10)$$

For the first mode contribution (j=1), the normalization is correct, and the reconstructed image is the same as in the ideal single-mode case. For other modes, the normalization does not correctly compensate for the contribution of the reference mode, and the holographic recorded field is multiplied by an erroneous factor of: $\frac{\left(E_{R,m}^{(j)}(x,y)\right)^*}{\left(E_{R,m}^{(1)}(x,y)\right)^*}$ (in addition to a different illumination of the object), which results in imaging artefacts in the reconstruction fields that are a coherent sum of the different modes contribution: $O_{reconst}(x,y) = \sum_j O^{(j)}(x,y,z_{prop})$.

Fig. S6 displays simulated reconstructed object fields for a USAF target for different relative amplitudes of second-order transverse mode excitation. As noted in the previous section, we estimate the energy of the second order mode in our experiments to be ~2% of the fundamental mode energy. Importantly, we note that the contributions of the higher-order modes is not a noise-like or background contribution, but a coherent term that contains imaging information. As such, it may be used for improved imaging by digitally taking into account the distribution of the reference field in the higher modes: $E_{R,m}^{(j)}(x,y)$, and the

difference in object illumination. This may be useful to improve the imaging NA and potentially the FoV, but will require a more involved reconstruction scheme, to simultaneously determine the object pattern and the modal excitation complex amplitudes.

Section S5: Distal Mirror Design

While to minimize footprint, the distal partially-reflecting mirror should ideally have a diameter not larger than the fiber diameter, in our proof-of-principle experiments the distal mirror was realized by a glass spacer and reflector, both made of two glass slides (with the reflector being coated at one facet). These had a diameter that was larger than the diameter of the fiber. However, a miniaturized version of the distal mirror can be realized by attaching a glass cylinder having the same diameter as the fiber to the fiber distal end, maintaining a minimal footprint. We display this design in Figure S9, which will be at the focus of future work. While such a distal mirror design is straightforward to implement, it can introduce additional spurious reflections from the lateral surfaces of the cylinder. Note that such spurious reflections are expected to be relatively weak due to the small index contrast between the cylindrical spacer and the surrounding medium. In addition, since light is both emitted and collected by a fundamental mode of the fiber cores, having a limited NA, the amplitude of the collected signal in the fundamental mode that results from reflections from the lateral surface of the cylindrical spacer is reduced twice: once in illumination and once in collection, as coupling back to the core depends on the angle of incidence.

Nonetheless, several solutions can be used to mitigate and further reduce such reflections: An anti-reflective coating can be applied to the lateral surface of the spacer. Alternatively, an absorptive/diffusive surface or a varying radial refractive index can be used to lower such reflections. In addition, a sufficiently short length for the cylindrical spacer length can be chosen, to ensure that higher angled beams do not reach the lateral surfaces (Fig.S9 dotted curve), at the price of a smaller imaging FOV.

Finally, if required, a computational approach that takes into account the total reflected reference field, including the residual spurious reflections, can be implemented in the reconstruction process.

Fig. S1

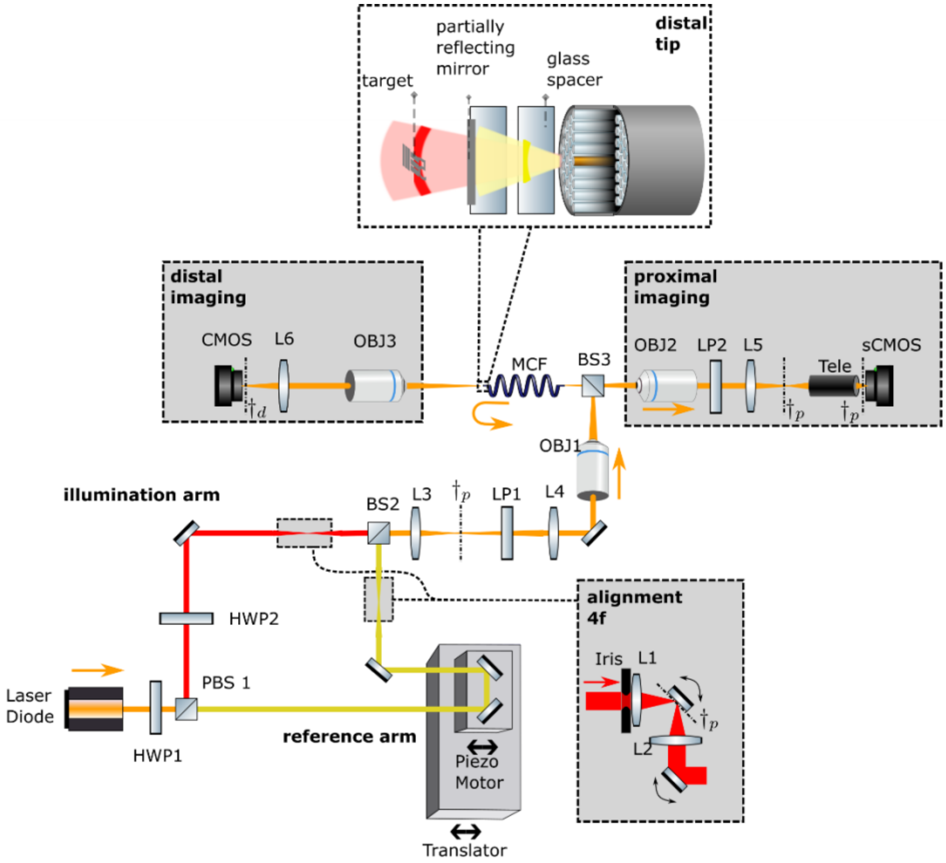

Fig. S1. Experimental setup. See section " Experimental Design " for complete description. HWP half-wave plate, PBS polarizing beam-splitter, BS beam-splitter, L lens, OBJ objective, MCF multicore fiber, Tele telescope, $\dagger_p, \dagger_d$ mark the proximal and distal conjugate planes, respectively.

Fig. S2

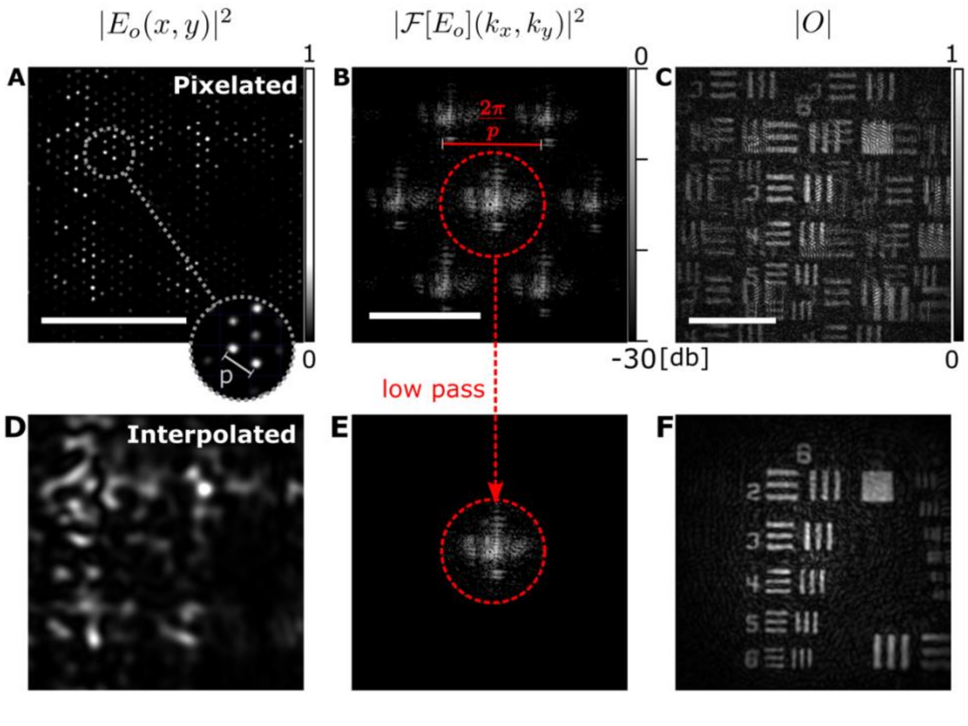

Fig. S2. Fourier Interpolation on ordered MCF (A) The measured distal field on a Schott fiber used throughout the article, shows the individual cores with a constant inter-core pitch, p, which perform effective spatial sampling of the field at a spatial frequency of $1/p$. (B) In the spatial frequency domain, replicas appear as result of the spatial sampling, as expected from the convolution theorem. The dashed red circle marks the spatial frequency range that is within the Nyquist sampling criterion ($|f| < \frac{1}{2p}$, i.e. $|k| < \frac{\pi}{p}$). (C) Reconstruction of the object directly from the measured field of (A), displays ghost replicas, due to the frequency-space aliasing. (D, E, F) show the same data, after low-pass filtering the aliased high spatial frequencies outside the dashed circle in (B). Scale bars: A, C - $100_{\mu m}$ B - $\frac{2\pi}{p}$

Fig. S3

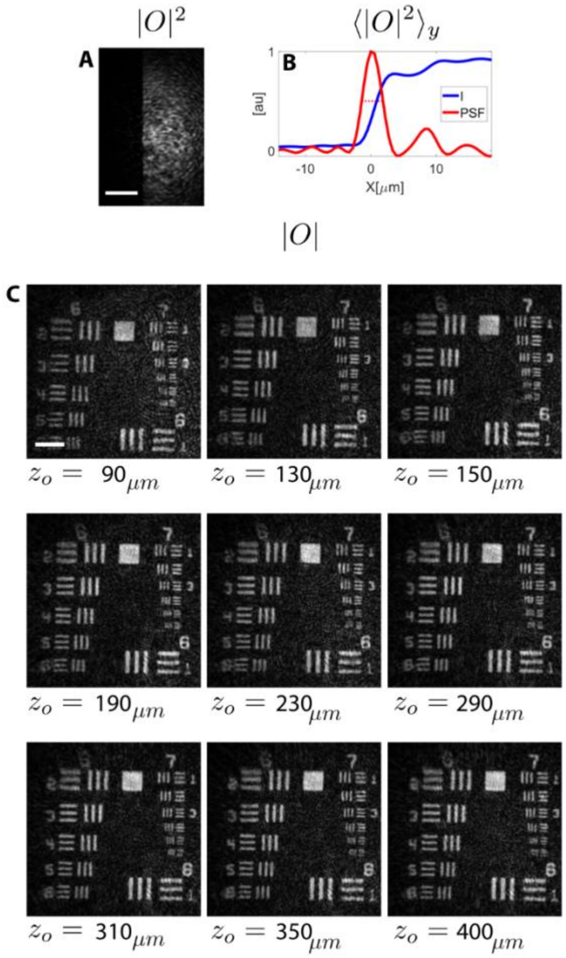

Fig. S3. Resolution and field-of-view characterization, detailed figure (**A**) a single image of the knife-edge mirror, using the edge of a USAF group 3 square, at a distance of $z_0 = 280\mu m$ (**B**) The mean cross section of (**A**)(blue), and the PSF (red) calculated as the spatial derivative of the cross section (red). The FWHM of the PSF, is indicated by the dashed line. (**C**) Imaging a USAF target at different depths, demonstrate the constant resolution and field of view. Scale bars: $50\mu m$

Fig. S4

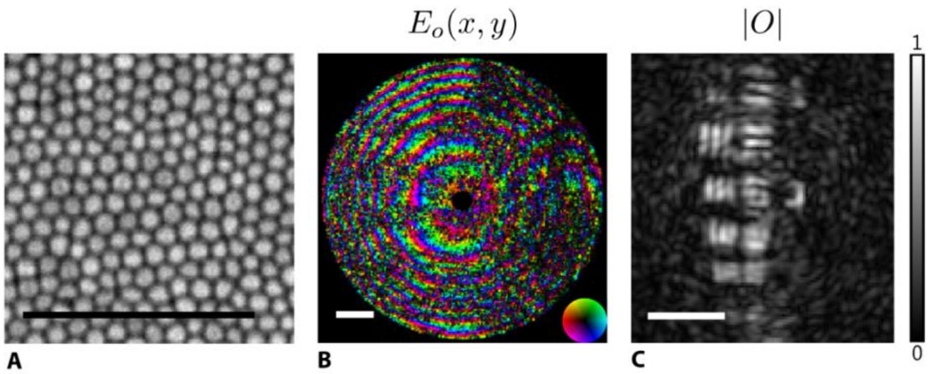

Fig. S4. Imaging using a disordered MCF (**A**) White light image of the FIGH-06-300S Fujikura fiber used (**B**) The retrieved distal field on the same fiber, when placing a reflective USAF resolution target at a distance of $z_0 = 100\mu m$ with the mirror placed at $z_m = 2_{mm}$. The cores are indiscernible due to binning of the camera pixels (**C**) The reconstructed object amplitude. Scale bars $40\mu m$.

Fig. S5

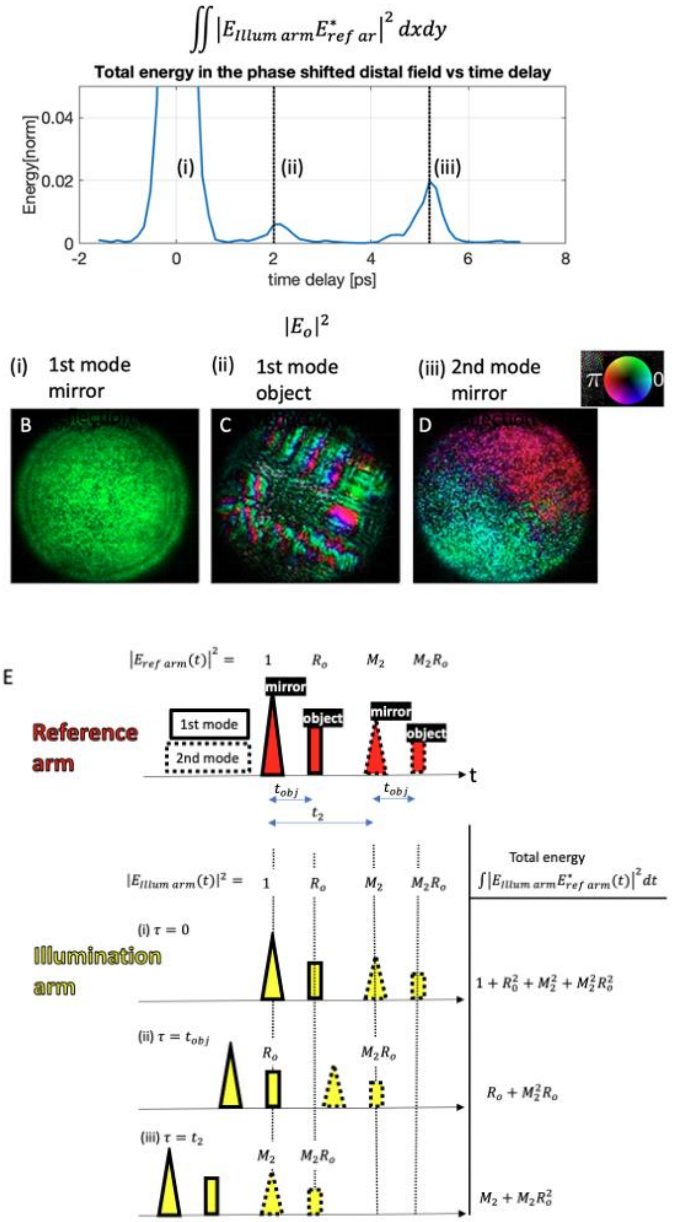

Fig S5: Effects of higher-order fiber-modes on time-gated holograms. (A) Total phase-shifted hologram energy vs delay introduced between the reference- and illumination-arms for a USAF-target placed at $z_{obj} = 205\mu m$. Three dominant peaks (i,ii,iii) are observed when the illumination delay matches either the zero relative delay (i), in which the reflected illumination ('object field') is dominated by the reflection from the distal mirror, the reconstructed hologram at this delay is a flat-phase and amplitude one (B). The second peak (ii) occurs when the delay matches the object distance $\frac{z_o n}{c}$, in which the object field is dominated by the first mode reflected from the target, and the reconstructed hologram is the object diffraction (C). The third peak (iii) occurs when the delay is equal to the difference between the second and first fiber mode arrival times, in which the distal object field is dominated by the reflection of the second mode from the distal mirror, and the reconstructed hologram is the diffraction of the second mode (D). Sketch (E) depicts the four delayed beams at the distal tip for each of the two arms, considering two fiber modes. Triangles and rectangles represent the mirror and object reflection respectively, solid and dashed lines represent the first and second mode respectively and red and yellow represent the reference and illumination arm respectively. The three peaks (i,ii,iii) are displayed as three different illumination delays. From the experimentally measured relative energies, we retrieve the approximated values for the reflected intensity from the object: $R_0 = 0.6\%$, and the second mode relative intensity: $M_2 = 2\%$

Fig. S6

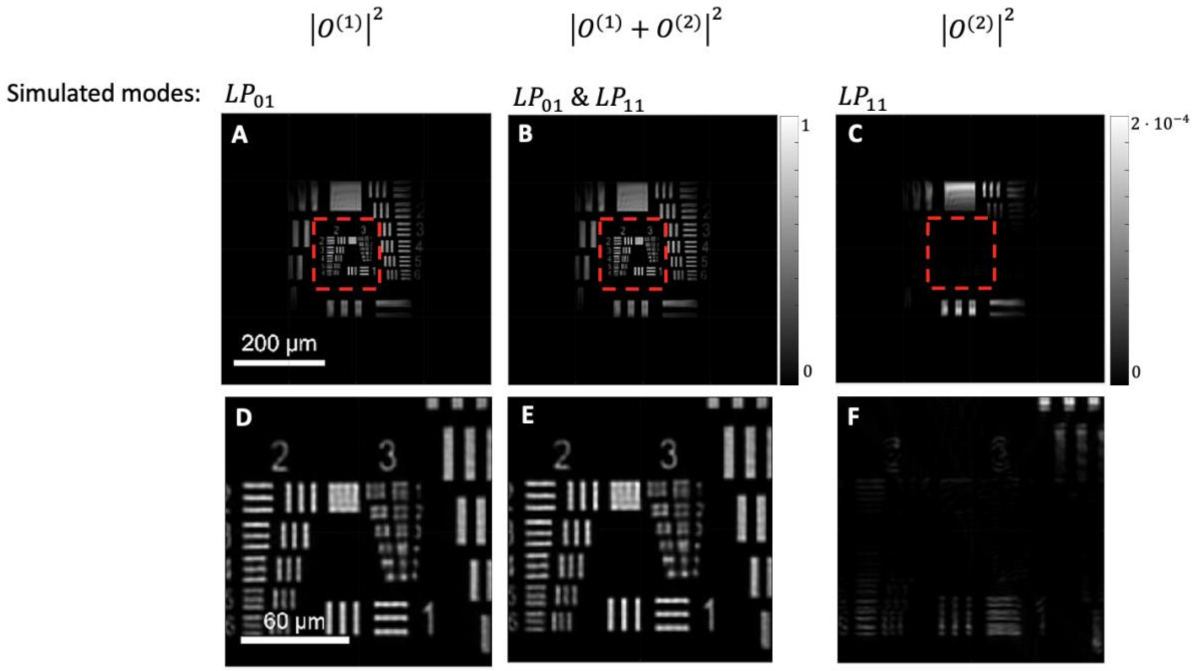

Fig S6: Simulated effect of higher-order fiber modes on object reconstruction. Numerical investigation of the reconstructed object field when the relative delay between the illumination- and reference arms matches the object distance (i.e. case (ii) of Fig.S5). (A) Assuming only the fundamental mode LP_{01} is excited in both reference and illumination arms. (B) Assuming 2% of the illumination energy excites the second (LP_{11}) fiber mode in both arms, as in our experiments (see Fig.S5). (C) Displays the simulated contribution of the second mode (LP_{11}), to the hologram. (D,E,F) are zoom-in on the dashed boxes in (A,B,C).

Fig. S7

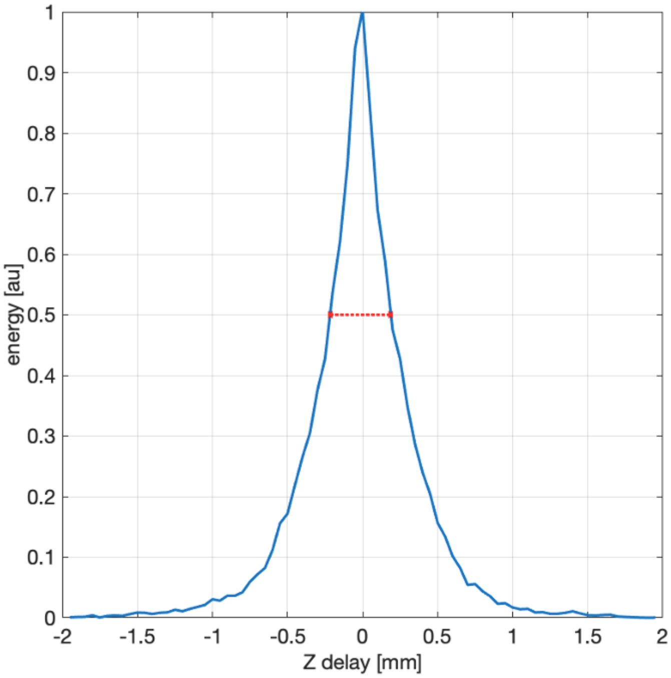

Fig S7: Measured Coherence function of the laser source. Total energy of phase-shifted hologram vs. the relative delay between the illumination and reference arms. The measurement was performed on the two beams directly at the output of the interferometer. The measured full-width at half max (FWHM) is $400\mu m \pm 50\mu m$ (red).

Fig. S8

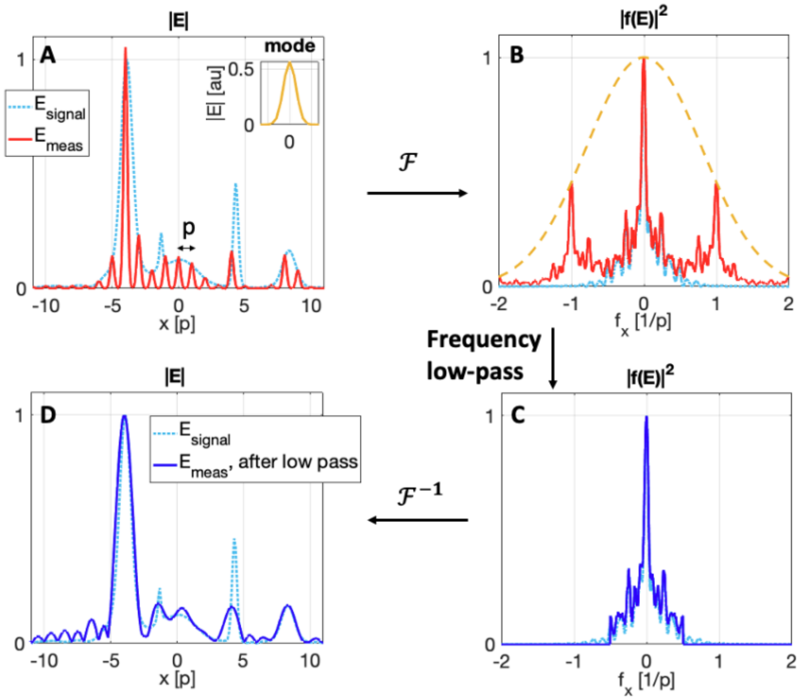

Fig S8: Fourier filtering (interpolation) process - 1D numerical example. (A) spatial distribution of the fields at the fiber facet: in the spatial domain, the holographically measured field through the MCF, $E_{meas}(x)$ (red) is the result of coupling of the incident field, $E_{signal}(x)$ (dashed cyan) to each of the MCF cores. The coupled field to each core is the overlap of the incident field with the fundamental mode of the core, $M(x)$ (inset, yellow). (B) The Fourier transform of the fields in (A): the spatial sampling by the ordered cores having a pitch, p, results in replicas of the original angular spectrum by spacing of 1/p. (C) Applying a low pass filter with a cutoff frequency of $f_{cutoff} = \frac{1}{2p}$ removes the spectral replicas aliased to high frequencies. The resulting low-pass filtered field (solid blue) closely resembles the original field angular spectrum (cyan). The remaining difference is the original spatial frequency content at $f_x > f_{cutoff}$ that either was not coupled to the fiber or filtered by the low pass filter, and aliased to $f_x < f_{cutoff}$. In our experiments the high spatial frequencies content is low due to the fiber parameters and illumination/detection geometry. (D) Inverse Fourier transform of the filtered field compared to the original field.

Fig. S9

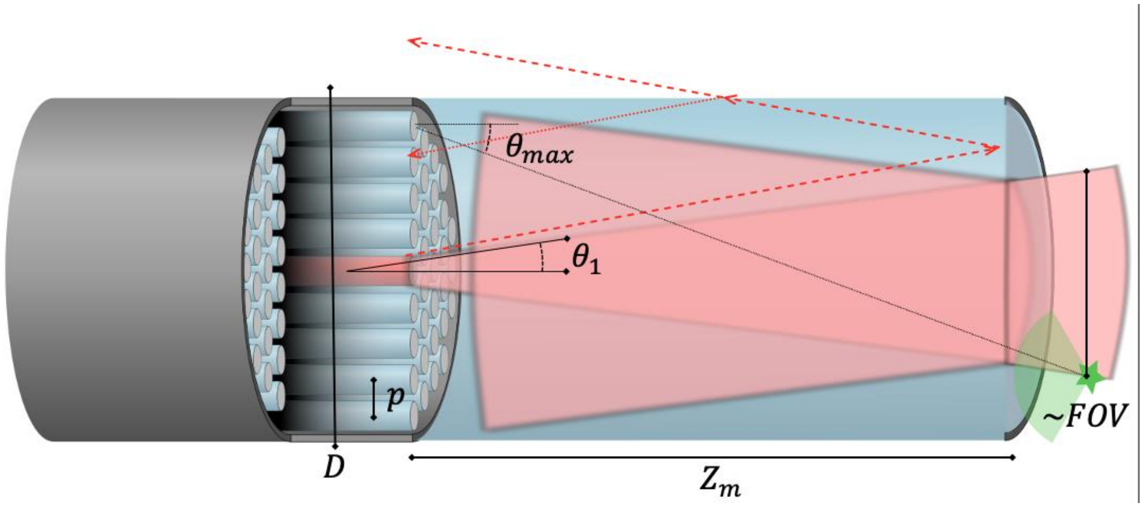

Fig. S9. Geometrical sketch of a proposed miniaturized partially-reflecting distal mirror composed of a transparent cylinder of diameter equal to the fiber diameter, D, and length Z_m. The reference beam cone marked in red, depicts the propagation of the fundamental mode, where most energy lies in angles limited by its numerical aperture: $NA = n \cdot sin\theta_1$. The mirror distance, Z_m, is optimally chosen such that the reflected reference beam covers the entire fiber facet. The red dashed lines illustrate the path of higher NA illumination, emitted via higher fiber modes or as found outside the FWHM of the fundamental mode. The dotted line illustrates a spurious, unwanted, weakly reflected reference illumination, which can be suppressed by proper choice of spacer materials and/or coating. θ_{max} depicts the maximum angle of incidence of detected light originating from objects points at the edge of the FOV: $\tan(\theta_{max}) \leq \frac{D}{2Z_m} + \tan(\theta_1)$.
