# Supplementary material for: Real-time holographic lensless micro-endoscopy through flexible fibers via fiber bundle distal holography
Source: Nat Commun. 2022 Oct 13;13:6055. doi: 10.1038/s41467-022-33462-y (PMC9563069; doi:10.1038/s41467-022-33462-y)
Supplement: Supplementary file 3 — Description of Additional Supplementary Files [file 41467_2022_33462_MOESM3_ESM.docx]

Supplementary files descriptions:

File Name: Supplementary Data 1 - reconstructionScript.zip
Description:

## Example MATLAB script and data set for reproducing results.

The MATLAB script and data set is an example that calculates the FiDHo reconstruction of a USAF reflective target, from 12 phase shifted camera frames and some system parameters.

File Name: Movie S1 - 60FPS imaging of a moving target.avi

Description:

Imaging a moving USAF target at 50 FPS. This movie displays the reconstruction of a moving target at 50 FPS. Individual frames are shown in Fig5A

File Name: Movie S2 - imaging while bending the fiber.avi

Description:

## Imaging a moving USAF target while bending the MCF.

This movie displays the reconstruction of a moving target simultaneously with the introduced bending. Fast dynamic bending within the three phase-shifting frames may impact the imaging quality, but the approach is insensitive to fiber orientation. Individual frames are shown in Fig 5B.
