# Supplementary figures and images for: Real-time holographic lensless micro-endoscopy through flexible fibers via fiber bundle distal holography
Source: Nat Commun. 2022 Oct 13;13:6055. doi: 10.1038/s41467-022-33462-y (PMC9563069; doi:10.1038/s41467-022-33462-y)

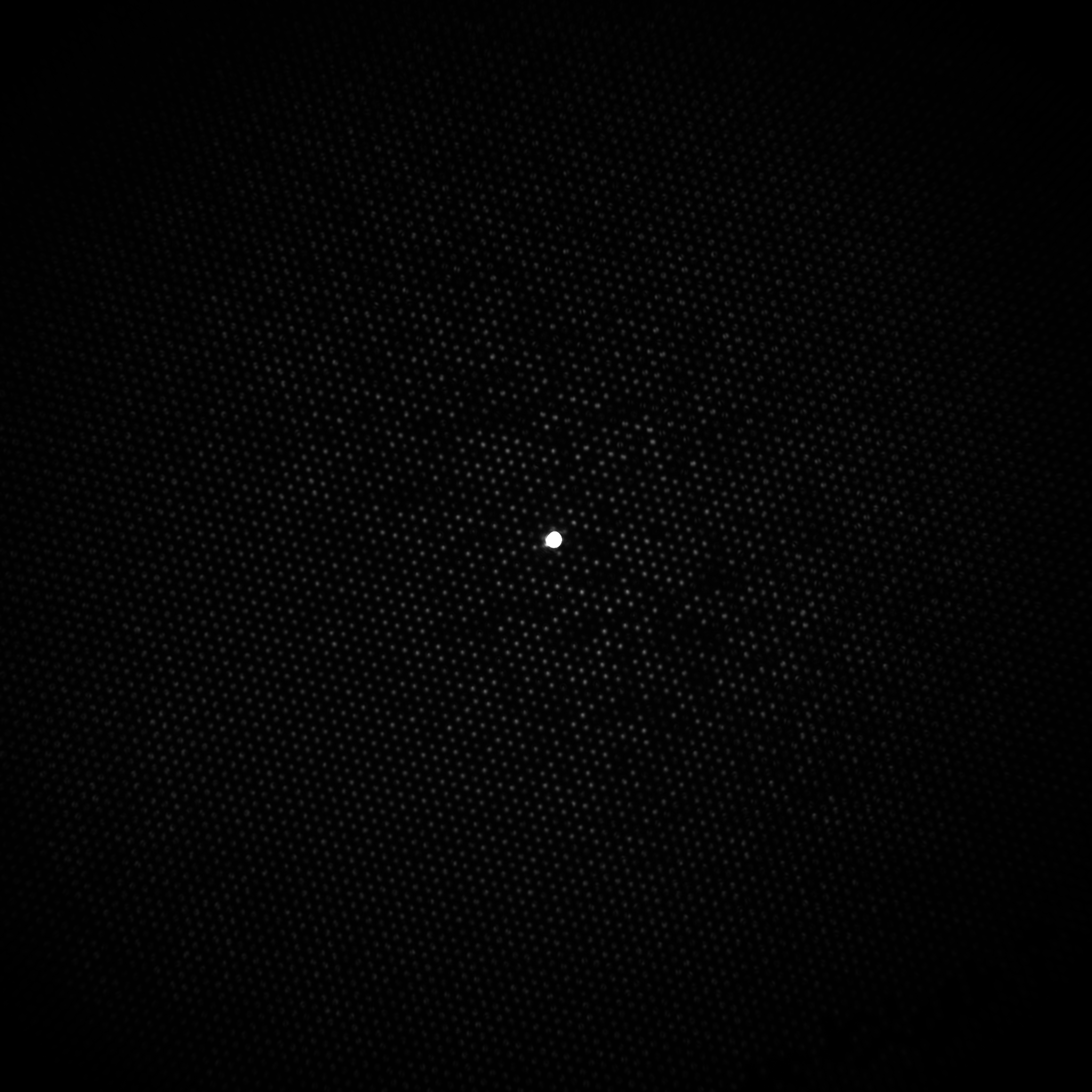

Supplement: Supplementary file 4 — Supplementary Data 1 [file 41467_2022_33462_MOESM4_ESM.zip › reconstructionScript/frame9.png]

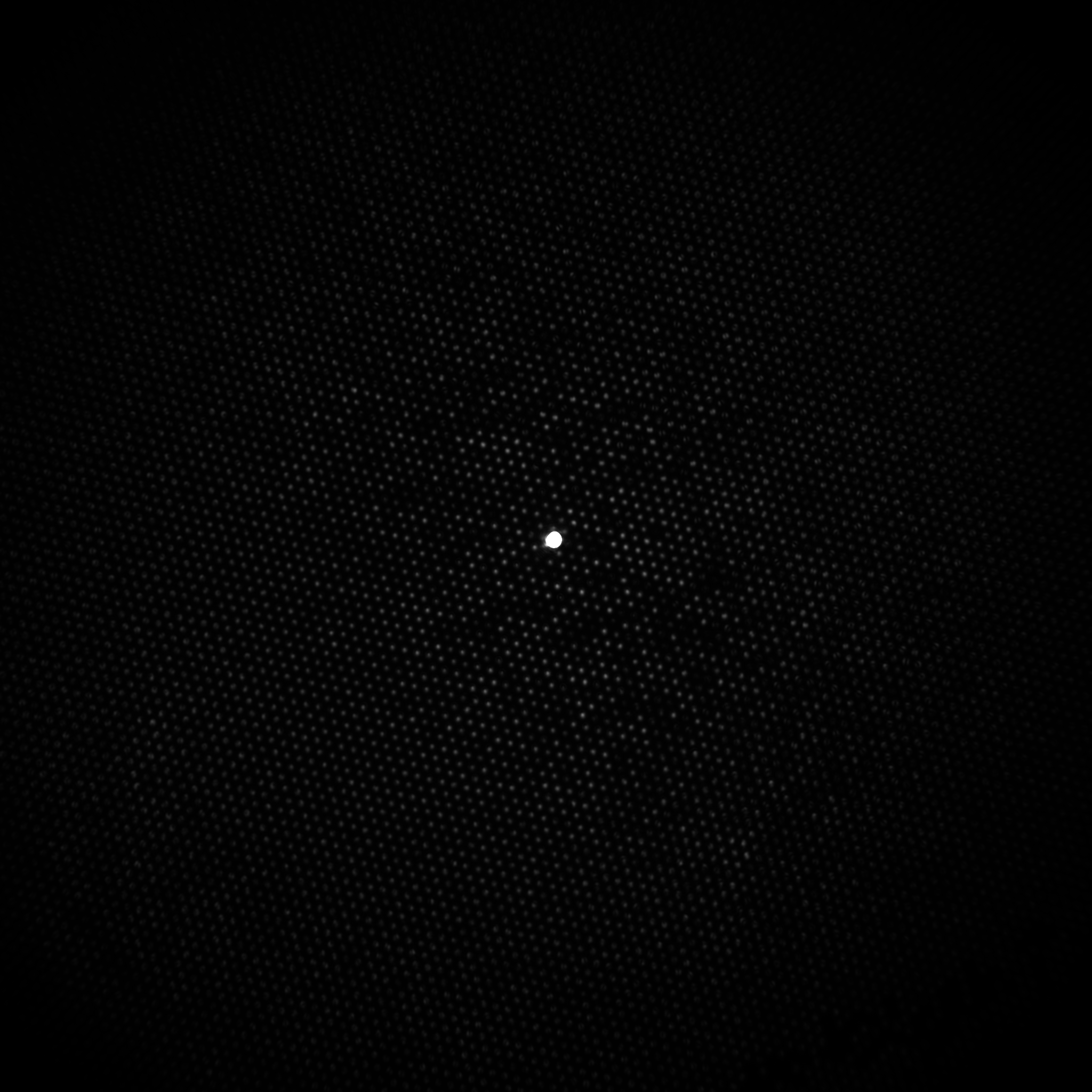

Supplement: Supplementary file 4 — Supplementary Data 1 [file 41467_2022_33462_MOESM4_ESM.zip › reconstructionScript/frame8.png]

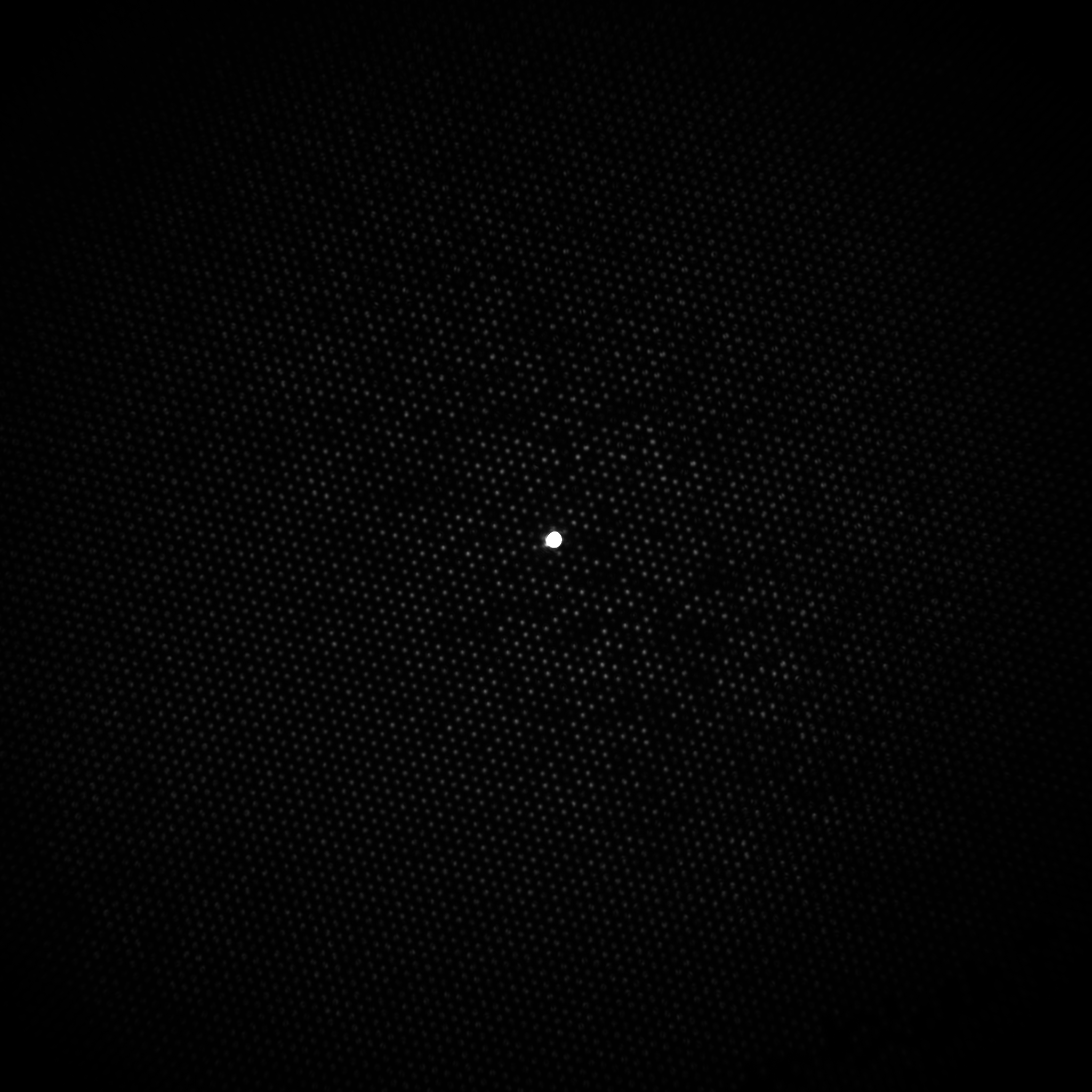

Supplement: Supplementary file 4 — Supplementary Data 1 [file 41467_2022_33462_MOESM4_ESM.zip › reconstructionScript/frame10.png]

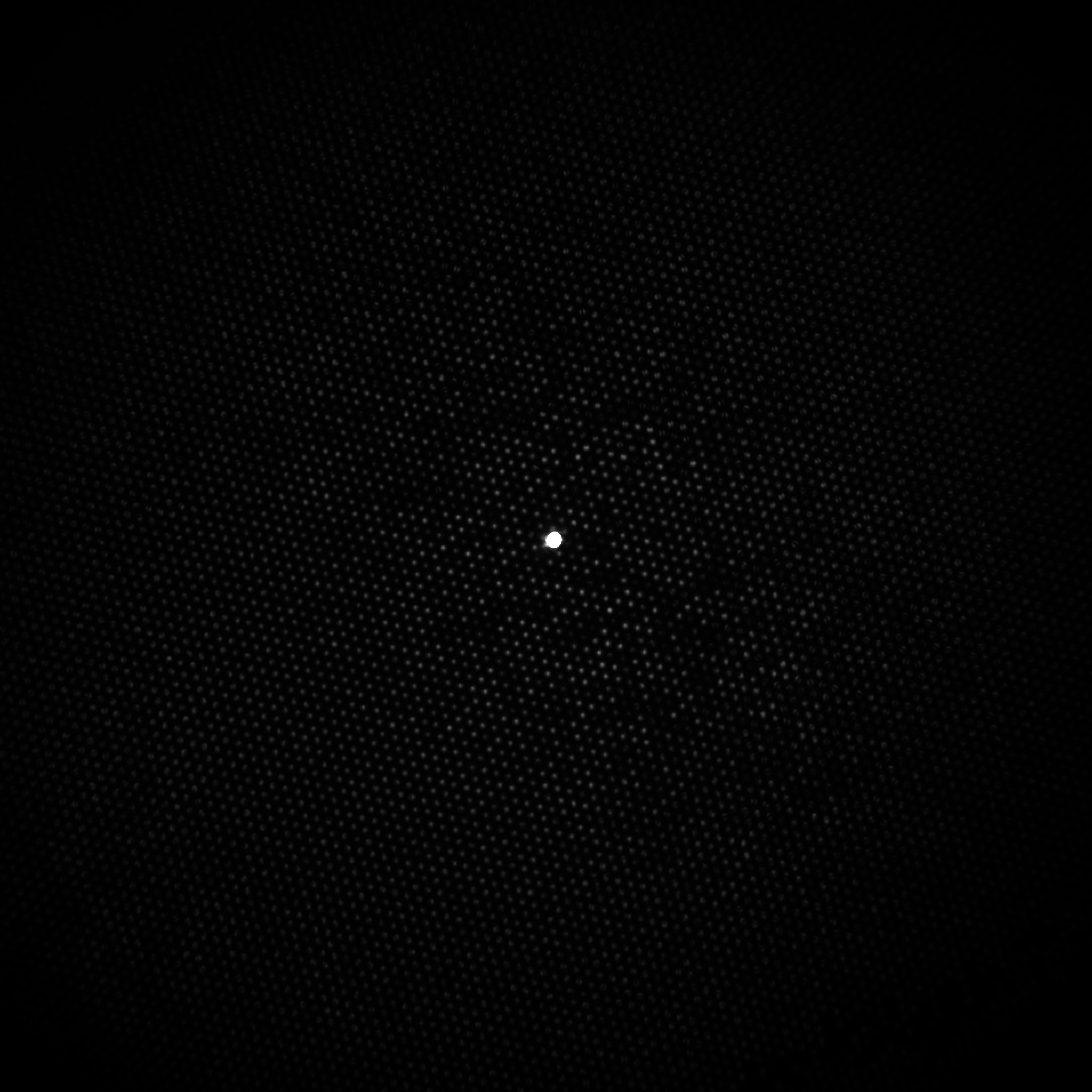

Supplement: Supplementary file 4 — Supplementary Data 1 [file 41467_2022_33462_MOESM4_ESM.zip › reconstructionScript/frame5.png]

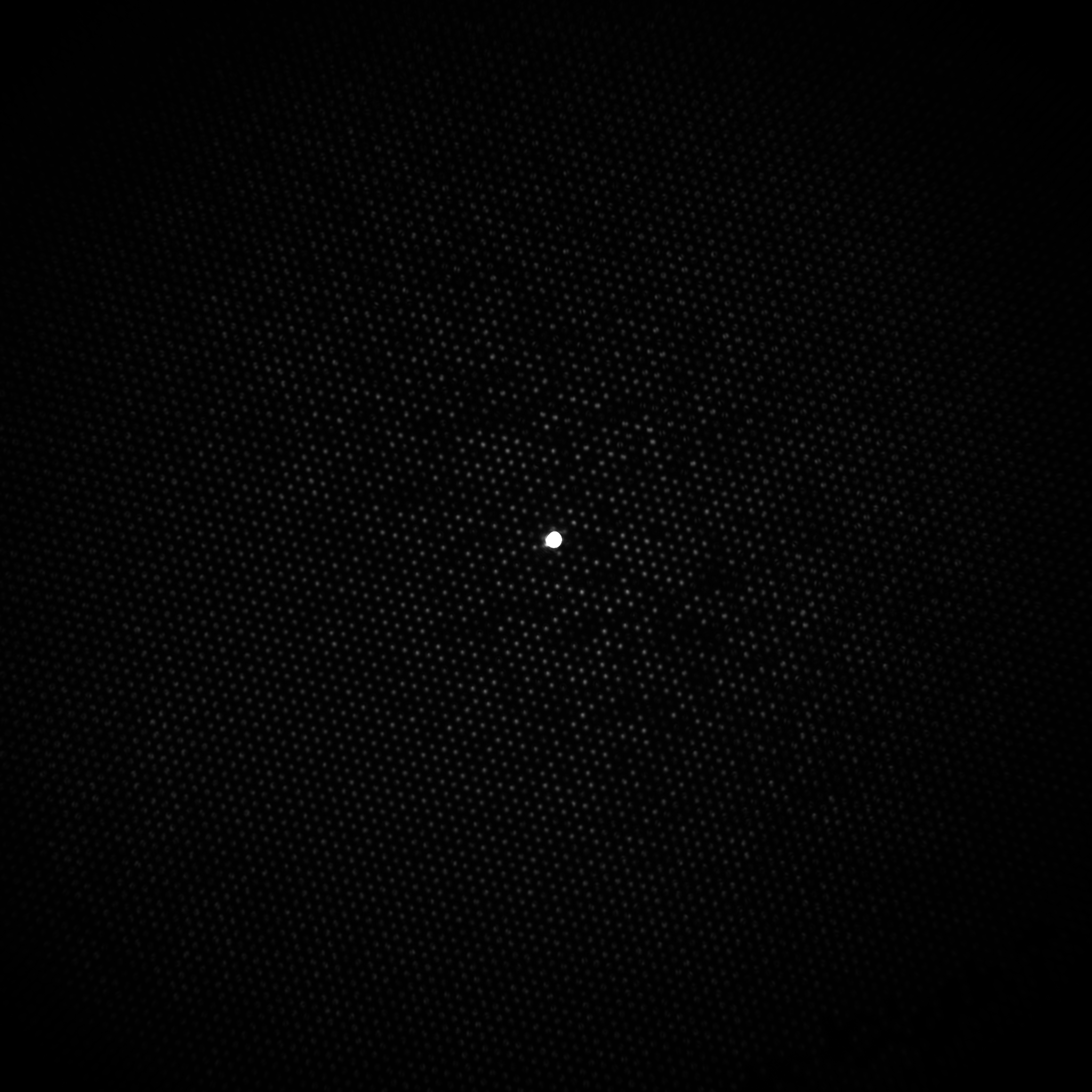

Supplement: Supplementary file 4 — Supplementary Data 1 [file 41467_2022_33462_MOESM4_ESM.zip › reconstructionScript/frame4.png]

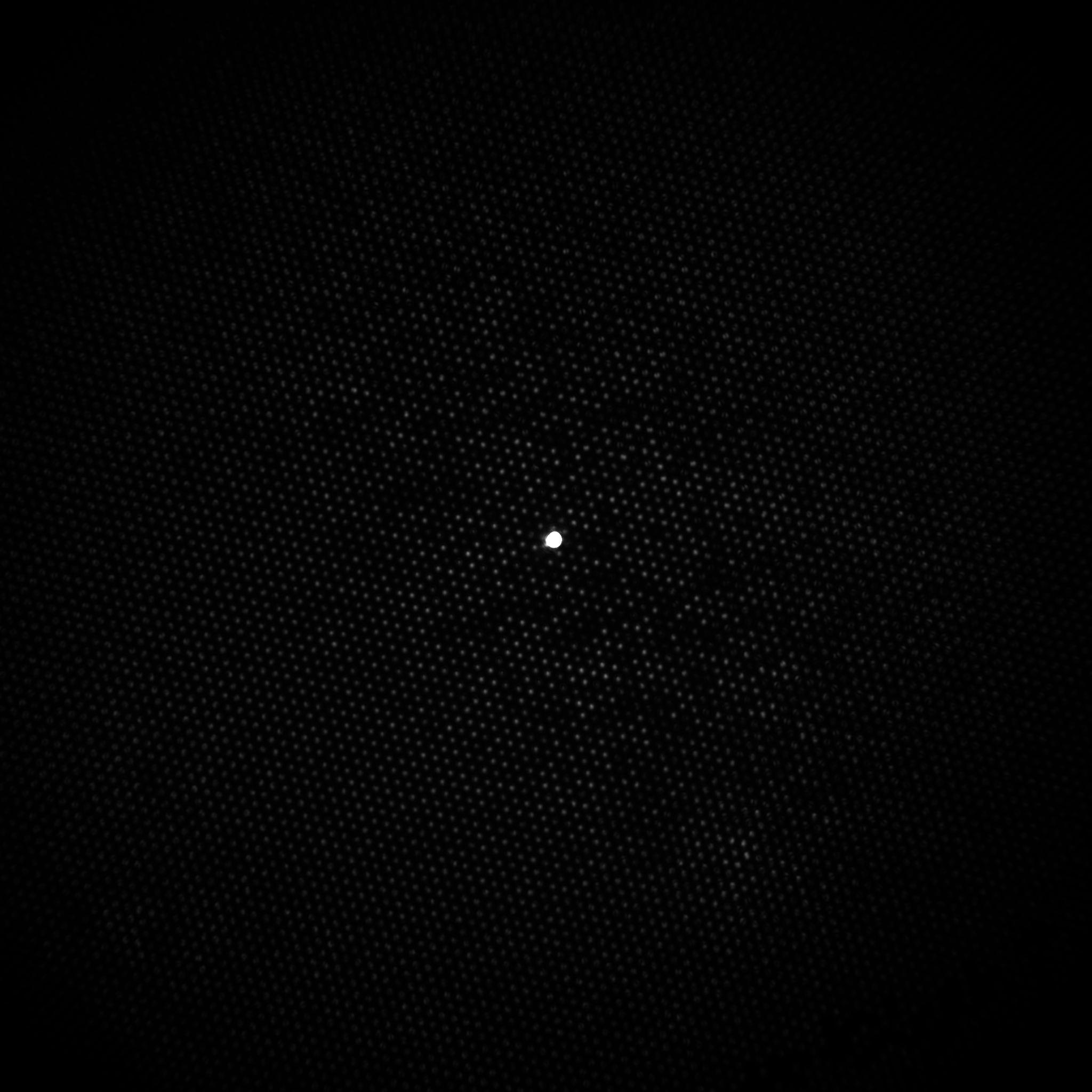

Supplement: Supplementary file 4 — Supplementary Data 1 [file 41467_2022_33462_MOESM4_ESM.zip › reconstructionScript/frame11.png]

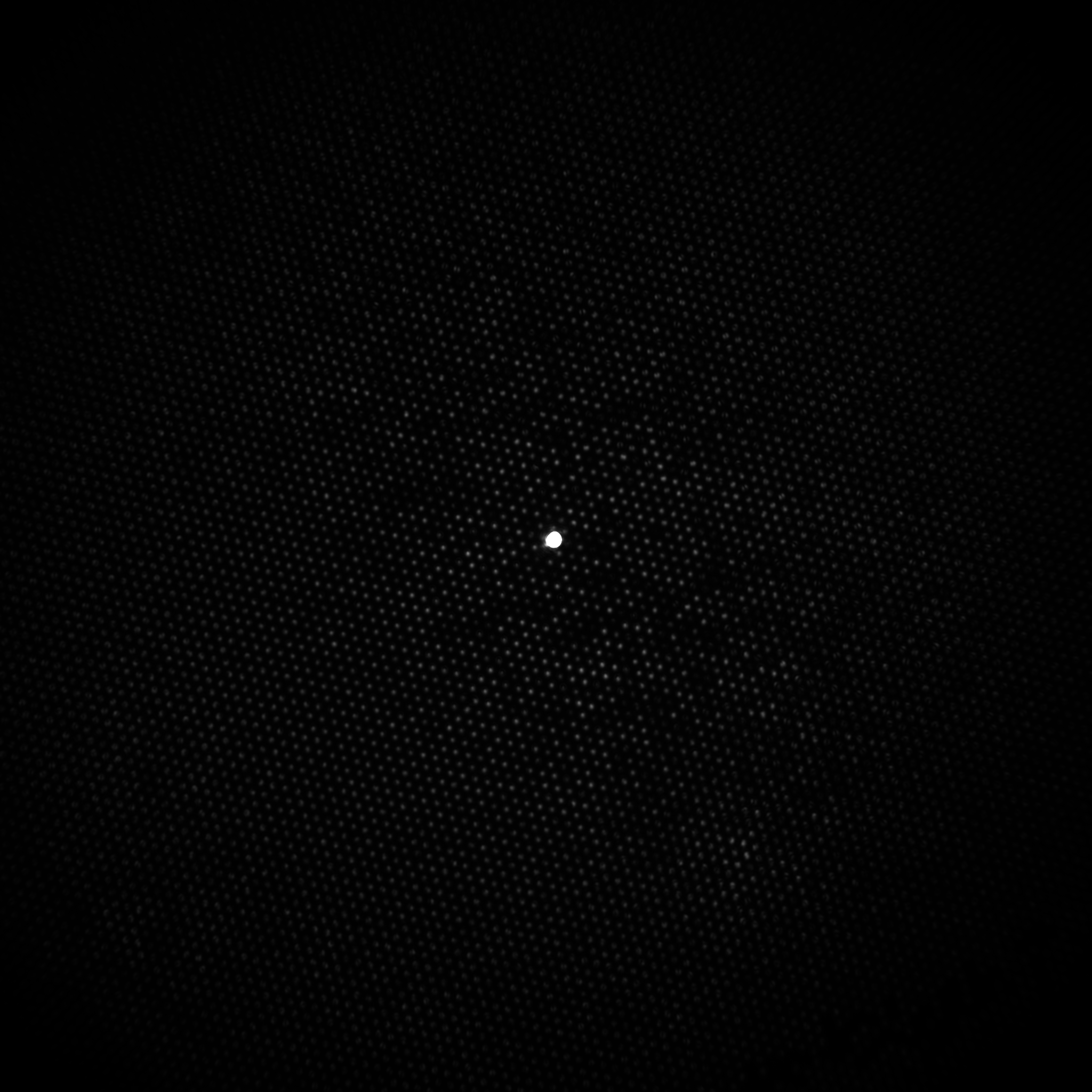

Supplement: Supplementary file 4 — Supplementary Data 1 [file 41467_2022_33462_MOESM4_ESM.zip › reconstructionScript/frame6.png]

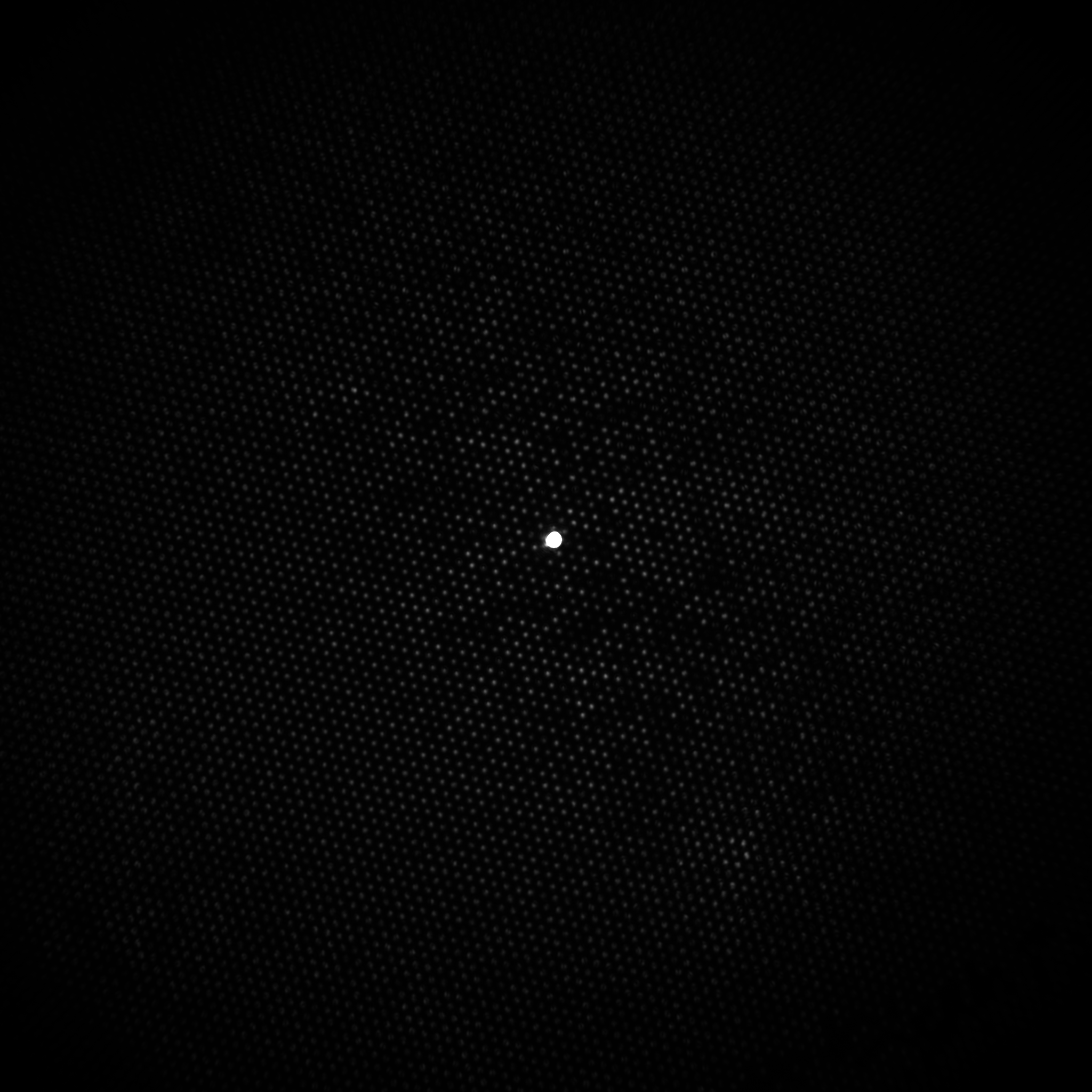

Supplement: Supplementary file 4 — Supplementary Data 1 [file 41467_2022_33462_MOESM4_ESM.zip › reconstructionScript/frame7.png]

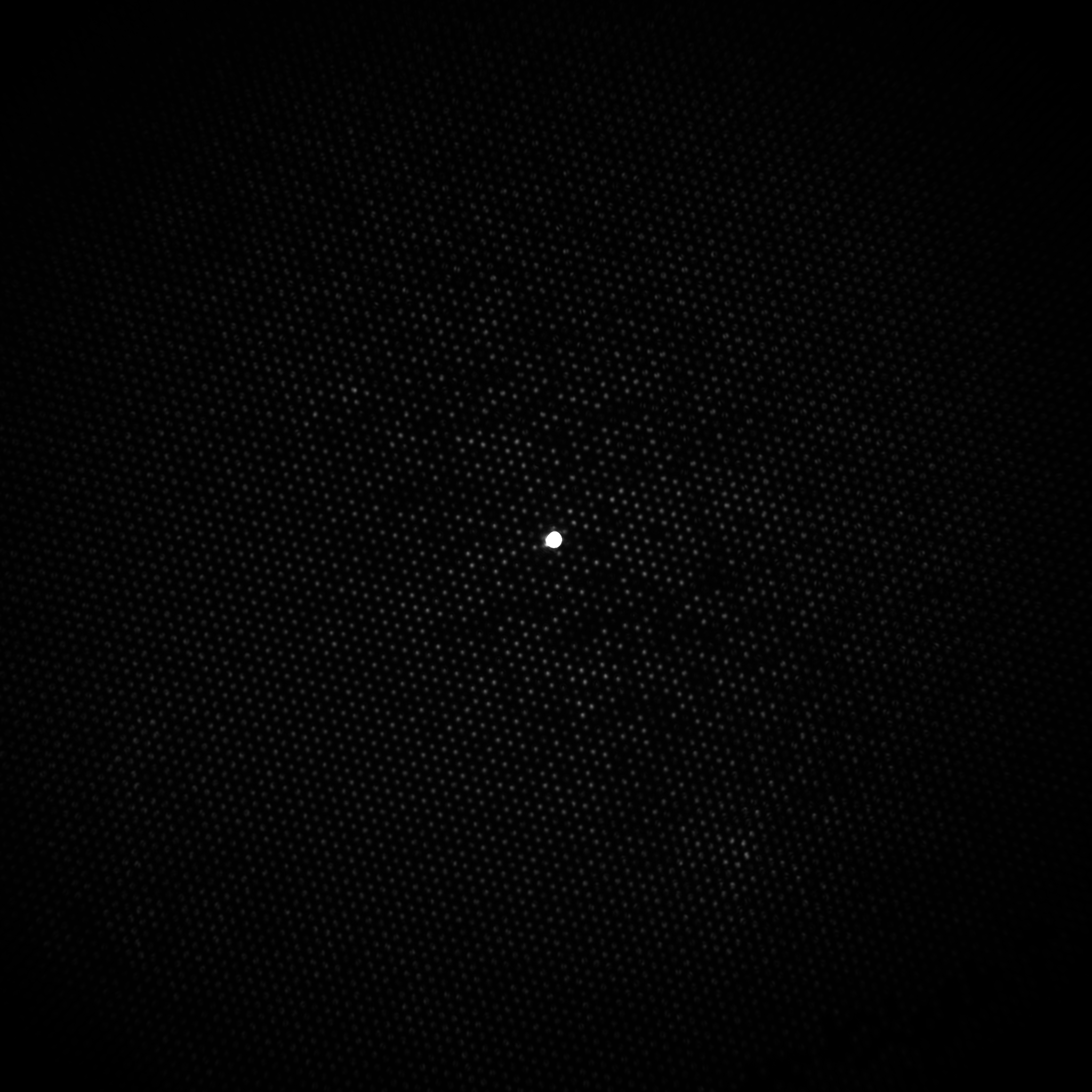

Supplement: Supplementary file 4 — Supplementary Data 1 [file 41467_2022_33462_MOESM4_ESM.zip › reconstructionScript/frame12.png]

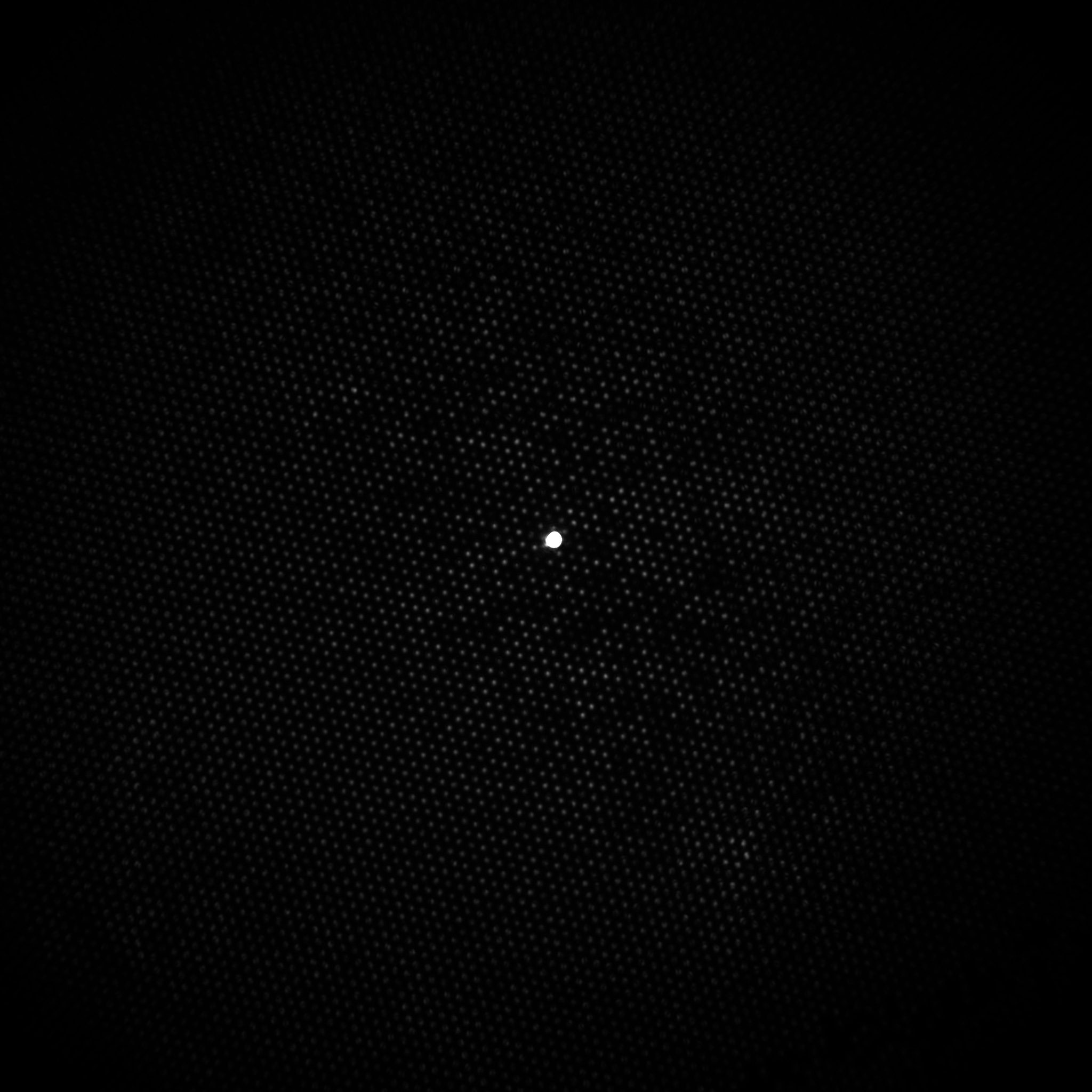

Supplement: Supplementary file 4 — Supplementary Data 1 [file 41467_2022_33462_MOESM4_ESM.zip › reconstructionScript/frame3.png]

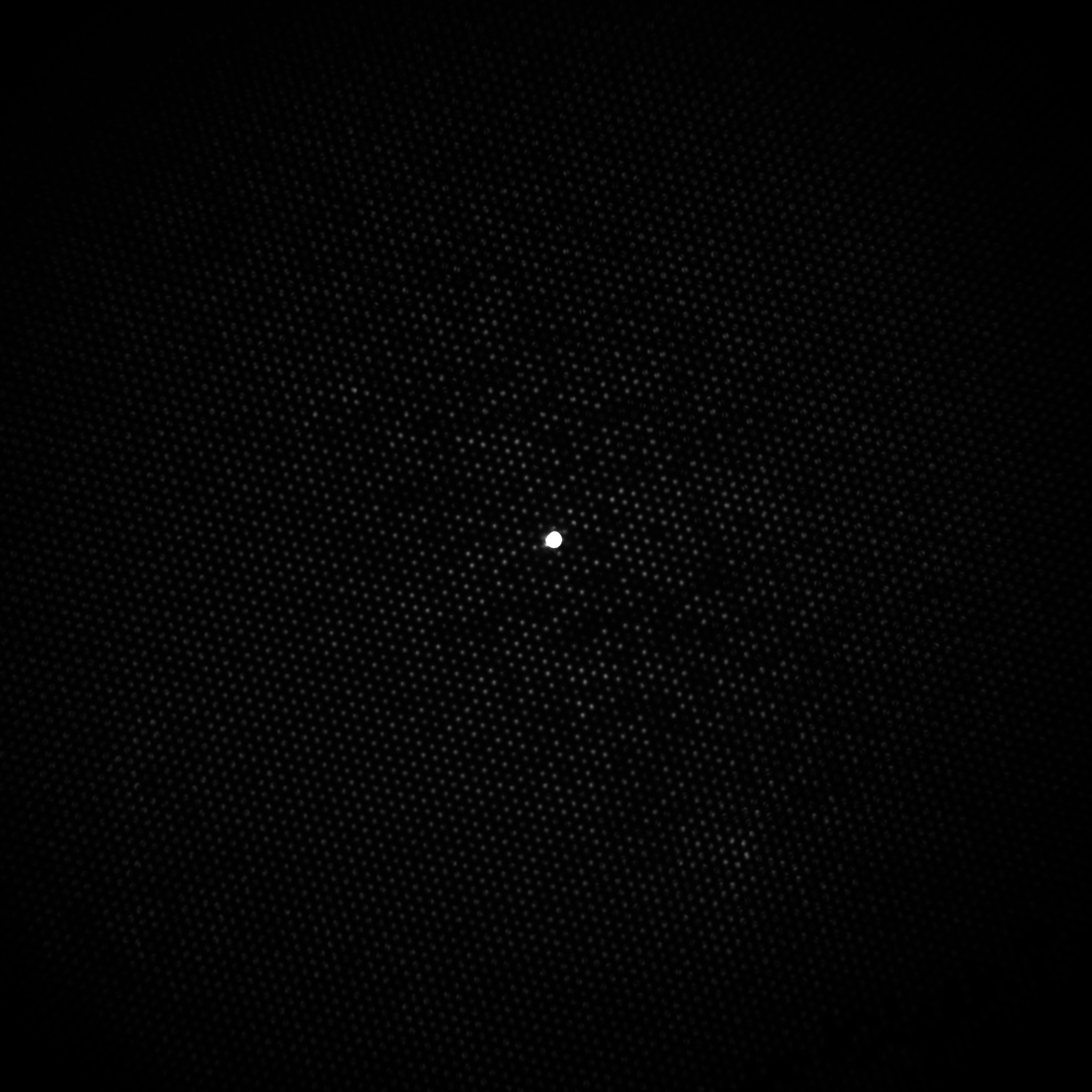

Supplement: Supplementary file 4 — Supplementary Data 1 [file 41467_2022_33462_MOESM4_ESM.zip › reconstructionScript/frame2.png]
